# Altered counts and mitochondrial mass of peripheral blood leucocytes in patients with chronic hepatitis B virus infection

**DOI:** 10.1111/jcmm.18440

**Published:** 2024-06-18

**Authors:** Ruo‐Ran Zhou, Ya‐Hui Song, Cheng‐Yu Xu, Ying‐Ying Zhang, Xiang‐Wei Wu, Lu Zhang, Xi‐Ni Luo, Han Zhao, Ming‐Ming Liu, Jun‐Chi Xu, Lin Wang, Zu‐Tao Chen, Qing‐Zhen Han

**Affiliations:** ^1^ Medical Center of Soochow University Suzhou Medical College of Soochow University Suzhou People's Republic of China; ^2^ Center of Clinical Laboratory and Translational Medicine The Fourth Affiliated Hospital of Soochow University, Suzhou Dushu Lake Hospital Suzhou People's Republic of China; ^3^ Infectious Disease Department The Fourth Affiliated Hospital of Soochow University, Suzhou Dushu Lake Hospital Suzhou People's Republic of China; ^4^ The Fifth People's Hospital of Suzhou Suzhou People's Republic of China; ^5^ Infectious Disease Department The First Affiliated Hospital of Soochow University Suzhou People's Republic of China

**Keywords:** hepatitis B virus, immune response, low mitochondrial membrane potential, mitochondrial mass, white blood cells

## Abstract

Hepatitis B virus (HBV) damages liver cells through abnormal immune responses. Mitochondrial metabolism is necessary for effector functions of white blood cells (WBCs). The aim was to investigate the altered counts and mitochondrial mass (MM) of WBCs by two novel indicators of mitochondrial mass, MM and percentage of low mitochondrial membrane potential, MMP^low^%, due to chronic HBV infection. The counts of lymphocytes, neutrophils and monocytes in the HBV infection group were in decline, especially for lymphocyte (*p* = 0.034) and monocyte counts (*p* = 0.003). The degraded MM (*p* = 0.003) and MMP^low^% (*p* = 0.002) of lymphocytes and MM (*p* = 0.005) of monocytes suggested mitochondrial dysfunction of WBCs. HBV DNA within WBCs showed an extensive effect on mitochondria metabolic potential of lymphocytes, neutrophils and monocytes indicated by MM; hepatitis B e antigen was associated with instant mitochondrial energy supply indicated by MMP^low^% of neutrophils; hepatitis B surface antigen, antiviral therapy by nucleos(t)ide analogues and prolonged infection were also vital factors contributing to WBC alterations. Moreover, degraded neutrophils and monocytes could be used to monitor immune responses reflecting chronic liver fibrosis and inflammatory damage. In conclusion, MM combined with cell counts of WBCs could profoundly reflect WBC alterations for monitoring chronic HBV infection. Moreover, HBV DNA within WBCs may be a vital factor in injuring mitochondria metabolic potential.

## 1 INTRODUCTION

Liver cancer is the sixth most common primary cancer in humans, usually progressing from liver inflammation and cirrhosis,^1^ and is also one of the most common causes of cancer mortality.^2^ Globally, hepatocellular carcinoma (HCC) is the dominant type of liver cancer accounting for approximately 75% of all liver cancers.^3^, ^4^ Moreover, hepatitis B virus (HBV)‐related cirrhosis resulted in an estimated 331,000 deaths in 2019, and it is estimated that the number of deaths from HBV‐related liver cancer in 2019 was 192,000, an increase from 156,000 in 2010.^5^ Nevertheless, on a global scale, in 2019, hepatitis B continued to pose a significant impediment to public health objectives, affecting 296 million individuals and resulting in approximately 820,000 fatalities,^6^, ^7^ and the highest incidence rates in the world are found in Asia and Africa.^8^ The two current antiviral drugs, nucleos(t)ide analogues and pegylated interferon‐alpha (PEG‐IFN‐α), rarely achieve functional cure of CHB (defined as loss of hepatitis B surface antigen [HBsAg]) and require lifelong treatment. Therefore, more and more studies have emphasized to take pioneering measures to delay the occurrence of HBV‐related liver injury.

HBV as an intracellular virus indirectly induces liver harm via aberrant immune reactions.^9^, ^10^ An increasing body of evidence supports this perspective, but recent research has predominantly focused on adaptive immune mechanisms, leaving the impact on innate immunity less explored. At the basis of immune cell proliferation and various other energy‐intensive processes lies mitochondrial metabolism.^11^ Recent investigations have revealed that HBV can incorporate its genetic material into mitochondrial DNA, thus impacting mitochondrial function.^12^

White blood cell (WBC) differential count is a feasible and easy method predicting viral infections.^13^ In this study, we combine WBC count and mitochondrial metabolism to investigate alterations in the numbers and cell metabolism on lymphocytes and innate immune cells, the latter including neutrophils and monocytes in chronic HBV infection. It employs two new‐style mitochondrial metrics: mitochondrial mass (MM) and low mitochondrial membrane potential (MMP^low^), indicating mitochondrial energy metabolism.^14^ The results reveal significant mitochondrial dysfunction in the lymphocytes and monocytes of individuals afflicted with chronic HBV infection developing no end‐stage cirrhosis or HCC, whereas decreased neutrophils and monocytes counts could predict liver fibrosis and inflammation by persistent HBV infection. Moreover, the presence of HBV DNA within these leucocytes emerges as pivotal factors influencing their mitochondrial metabolism and functionality. Consequently, in addition to adaptive immune response, MM and the number of innate immune cells, such as neutrophils and monocytes have also been altered in peripheral blood of patients. with chronic HBV infection, although they have not yet progressed to end‐stage liver disease.

## 2 MATERIALS AND METHODS

### 2.1 Study population

During July 2023 and February 2024, 98 adults were enrolled in outpatient service or physical examination departments from three medical institutions in Suzhou, Jiangsu, China: The Fourth Affiliated Hospital of Soochow University, The First Affiliated Hospital of Soochow University and The Affiliated Infectious Diseases Hospital of Soochow University. Forty‐two healthy people with no HBV carriage or close contacts as control were undergoing no known diseases identified by laboratory tests, imaging examinations and clinical manifestations by healthcare doctor enquiries. Fifty‐six patients undergoing chronic HBV infection but no end‐stage liver disease were diagnosed according to the definition of HBsAg, or HBV DNA seropositive, or both for ≥6 months.^15^ The exclusion criteria were as follows: (i) patients aged <18 years; (ii) patients with malignant tumours; (iii) patients who underwent organ transplantation or were long‐term users of drugs that affect immune function (such as adrenocortical hormone); (iv) patients with end‐stage liver disease (cirrhosis, liver cancer, to avoid confounding immune alterations^16^, ^17^); and (v) undergoing PEG‐IFN‐α antiviral therapy (to avoid confounding effects of PEG‐IFN‐α on the immune system^18^, ^19^); (vi) common diseases such as diabetes and hypertension. Additionally, the matching of controls to cases was executed with the controls (median age 36 with an interquartile range of 17) and CHB patients (median age 38 with an interquartile range of 19) closely aligned in age, and similarly, the gender ratio was comparably balanced between the groups, with 27 males to 15 females in the control group and 34 males to 22 females in the CHB group.

### 2.2 WBC count and mitochondrial indicator measurements by flow cytometry

The peripheral blood sample was collected in tubes coated with EDTA‐k anticoagulant and detected within 48 h by flow cytometry. The detection parameters include percentage, absolute cell count, MM and MMP^low^ of lymphocytes, neutrophils and monocytes. Three types of WBCs are mainly classified based on their size and internal particles indicated by forward scatter and side scatter from flow cytometry (FACSCantoII, BD, USA). At least 20,000 cells were collected from each sample. Absolute counts were ×(10^6^/L) and proportion in percentage was calculated in total WBCs. The MFI (median fluorescence index) of mitochondria was used to calculate mitochondria mass, and it was detected by the APC channel of the flow cytometer. The percentage of low mitochondrial membrane potential (MMP^low^%) was clustered by mitochondrial peculiar marker MitoDye (UB Biotechnology Co., LTD, Zhejiang, China) and gate the percentage of low population cells (ratio value). As Figure S1 shows, lymphocyte counts and mitochondrial mass (MM and MMP^low^%) were determined by flow cytometry.

### 2.3 Conventional measurements

Virological markers of HBV in serum were measured prospectively during the clinical study, including HBV DNA, HBsAg and hepatitis B e antigen (HBeAg). Serum HBV DNA was measured using a real‐time fluorescence quantitative PCR detection kit (Sansure, China) on Roche480II (Roche Diagnostics, USA), which has a lower limit of detection (LOD) of 60 IU/mL. Serum HBsAg and HBeAg were measured using a commercial ChemiLuminescence kit (Abbott, USA) with a LOD of 0.05 IU/mL HBsAg, positive HBeAg as >1 S/CO. Serum alanine aminotransferase and aspartate aminotransferase were determined by an automatic biochemical analyser platform of Cobas8000 (Roche Diagnostics, USA).

### 2.4 Detecting HBV DNA within WBCs

A 2‐mL ACK lysis buffer was used to destroy red blood cells of 1 mL peripheral blood at room temperature for 5 min. Residual WBCs were collected by centrifugation (1000 rpm, 8 min) and washed twice with sterile saline. Collected WBCs were destroyed by a cell lysate (BioPerfectus, Jiangsu, China) to liberate the cellular nucleic acids through 100°C heating for 10 min. The mixture was centrifuged at 12, 000 rpm for 5 min and the supernatant was used for subsequent nucleic acid testing. Quantitative real‐time fluorescent PCR (qRT‐PCR) was performed on LightCycler480II Real‐Time PCR Detection System (Roche, USA) using a commercial HBV DNA test kit (Sansure Biotech Inc, HuNan, China).

### 2.5 Statistical analysis

The statistical analysis was performed using GraphPad Prism 9 and SPSS 26 software. Continuous variables were summarized as the mean (SD) or median (interquartile range [IQR]) and compared using the *t*‐test or Mann–Whitney test. Categorical variables were summarized as numbers (percentages) and compared using the chi‐squared test. The area under the curve (AUC) of the receiver operating characteristic (ROC) curve was calculated to assess the performance in the prediction of disease classification. The Youden index was calculated based on the ROC to help set an appropriate cut‐off value. All *p* values were two‐tailed, and values <0.05 are considered statistically significant.

## 3 RESULTS

### 3.1 Clinical characteristics of patients with chronic HBV infection

As shown in Table 1, this study enrolled 42 healthy individuals and 56 patients with chronic HBV infection, with no significant differences in median age and sex ratio between the two groups. All HBV‐infected patients were positive for HBsAg, of which 22 were positive for HBV DNA and 21 positive for HBeAg. Among the 56 patients with chronic HBV infection, 16 progressed to chronic hepatitis B (CHB) with detectable liver injury caused by persistent HBV infection for ≥6 months. Thirty‐six patients had previously or were currently undergoing antiviral treatment with nucleos(t)ide analogues, and the 12 patients with unknown treatment have undergone no PEG‐IFN‐α therapy and no other definite antiviral therapy information by nucleos(t)ide analogues during the whole course of HBV infection.

**TABLE 1:** Clinical characteristics of patients with chronic HBV infection.

Characteristics	Healthy control group (*n* = 42)	Chronic HBV infection group (*n* = 56)
Median age (IQR)	36 (17)	38 (19)
Gender ratio (M:F)	27:15	34:22
Discovery time of hepatitis B/year, *n*		
<5 years	–	13
5–10 years	–	3
>10 years	–	22
Unknown	–	18
Serum HBV DNA (+), *n* (%)	–	22 (39.29)
HBsAg(+)	–	60 (100)
HBeAg(+)	–	21 (37.50)
WBC HBV DNA(+)	–	18 (32.14)
Liver damage, *n* (%)		
No significant abnormalities	–	40 (71.43)
Liver with inflammatory necrosis or fibrosis	–	16 (28.57)
Treatment status, *n* (%)		
Nucleos(t)ide analogue treatment	–	36 (64.29)
No treatment	–	8 (14.29)
Unknown	–	12 (21.43)

Abbreviations: HBeAg, hepatitis B e antigen; HBsAg, hepatitis B surface antigen; HBV, hepatitis B virus; IQR, interquartile range; WBC, white blood cell.

### 3.2 Alterations in peripheral blood leucocyte classification and mitochondrial activity in patients with chronic HBV infection

As shown in Table 2, compared to the healthy control group, the counts of lymphocytes (*p* = 0.034) and monocytes (*p* = 0.003) in the HBV infection group showed a declining trend. Regarding mitochondrial function, lymphocytes and monocytes presented significant alterations in HBV‐infected patients. Specifically, lymphocytes demonstrated a significant reduction in MM (*p* = 0.003) and a significant increase in low MMP^low^% (*p* = 0.002); monocytes exhibited only a decreased MM (*p* = 0.005).

**TABLE 2:** Variations in WBC count and mitochondrial activity in patients with chronic HBV infection.

Characteristics	Healthy control group (*n* = 42)	Chronic HBV infection group (*n* = 56)	*t* ^a^/*z* ^b^	*p* value
Lymphocyte count (10^6^/μL)	1945.57 ± 749.20	1632.55 ± 509.10	*t* = −2.150	0.034
Lymphocyte percentage	40.19 ± 11.71	38.31 ± 11.49	*t* = −0.797	0.428
MM of lymphocyte	1.56 (0.94)	1.09 (1.01)	*z* = −2.879	0.003
MMP^low^% of lymphocyte	43.95 (24.44)	54.98 (34.43)	*z* = −2.426	0.002
Monocyte count (10^6^/μL)	276.34 ± 182.22	187.12 ± 133.40	*t* = −2.800	0.003
Monocyte percentage	6.01 ± 1.40	5.52 ± 1.85	*t* = −1.439	0.153
MM of monocyte	3.95 (4.43)	2.85 (2.79)	*z* = −2.513	0.005
MMP^low^% of monocyte	6.36 (12.82)	7.23 (14.05)	*z* = −0.133	0.894
Neutrophil count (10^6^/μL)	2259.29 ± 1815.80	1745.66 ± 1249.09	*t* = −1.658	0.100
Neutrophil percentage	52.54 ± 12.34	52.12 ± 13.36	*t* = −0.157	0.876
MM of neutrophil	2.51 (2.58)	1.93 (2.27)	*z* = −1.802	0.072
MMP^low^% of neutrophil	14.65 (22.00)	12.32 (17.89)	*z* = −0.158	0.875

^a^ *t*‐test was used for normally distributed data.

^b^ Mann–Whitney test was used for non‐normally distributed data.

Abbreviations: HBV, hepatitis B virus; MM, mitochondrial mass; MMP^low^%, percentage of low mitochondrial membrane potential; WBC, white blood cell.

### 3.3 High‐risk factors leading to alterations in WBC mitochondrial function and counts

**FIGURE 1:**
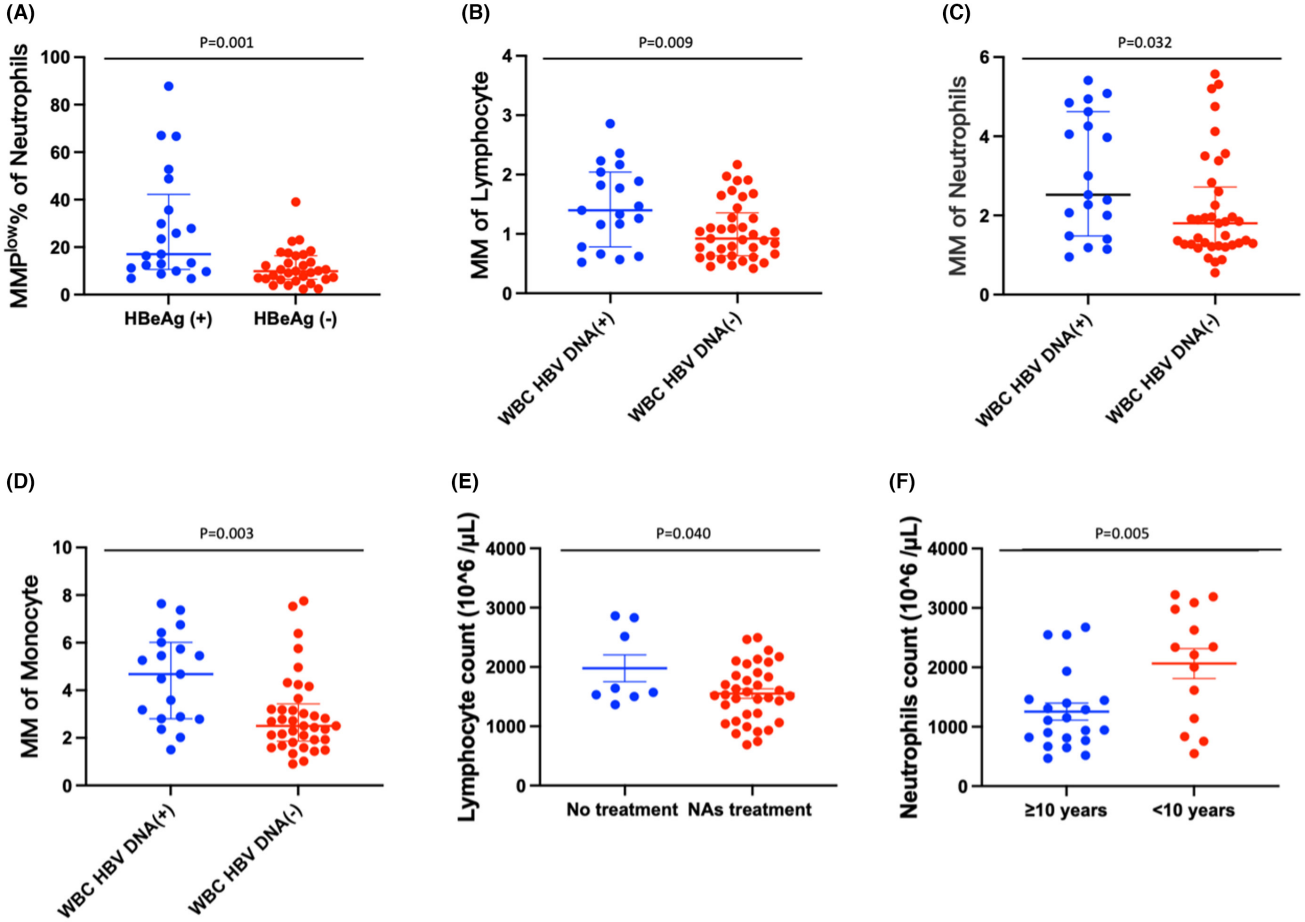
Significantly different distributions of peripheral blood white blood cells in different hepatitis B populations. Different MMP^low^ % of neutrophils between HBeAg(+) and HBeAg(−) in patients with chronic HBV infection (A), altered MM of lymphocytes (B), neutrophils (C) and monocytes (D) between patients with HBV DNA‐positive or ‐negative WBC. Different lymphocyte count in patients who underwent no treatment or treatment with nucleos(t)ide analogue (NA) drugs (E). Different neutrophil count in patients infected for over 10 years and less than 10 years (F). HBeAg, hepatitis B e antigen; HBsAg, hepatitis B surface antigen; HBV, hepatitis B virus; MM, mitochondrial mass; MMP^low^%, percentage of low mitochondrial membrane potential; WBC, white blood cell.

All the enrolled patients with chronic HBV infection were grouped according to the seropositivity of HBeAg, DNA and the presence of HBV DNA within WBCs. As shown in Figure 1, the positivity of serum HBeAg increased MMP^low^% of neutrophils (*p* = 0.001). However, positivity for serum HBV DNA did not exhibit a significant impact on the count or mitochondrial metabolism. Moreover, the presence of HBV DNA within WBCs significantly increased the MM of lymphocytes (*p* = 0.009), neutrophils (*p* = 0.032) and monocytes (*p* = 0.003). Considering all the enrolled patients with positive HBsAg for more than half a year indicating persistent HBV infection, HBsAg may induce other alterations in lymphocyte count, monocyte count and lymphocyte MMP^low^% as listed in Table 1. Overall, among viral components, HBV DNA within WBCs may be a vital factor affecting the mitochondria metabolic potential of WBCs indicated by MM; HBeAg and HBsAg may affect MMP^low^% of neutrophils presenting its instant activity; HBsAg may lead to extensive affection on WBC counts.

Additionally, treatment with nucleos(t)ide analogues significantly reduced the total count of lymphocytes (*p* = 0.040). With prolonged HBV infection, neutrophil count decreased significantly (*p* = 0.005).

### 3.4 Neutrophil and monocyte counts predicting liver damage by chronic HBV infection

**FIGURE 2:**
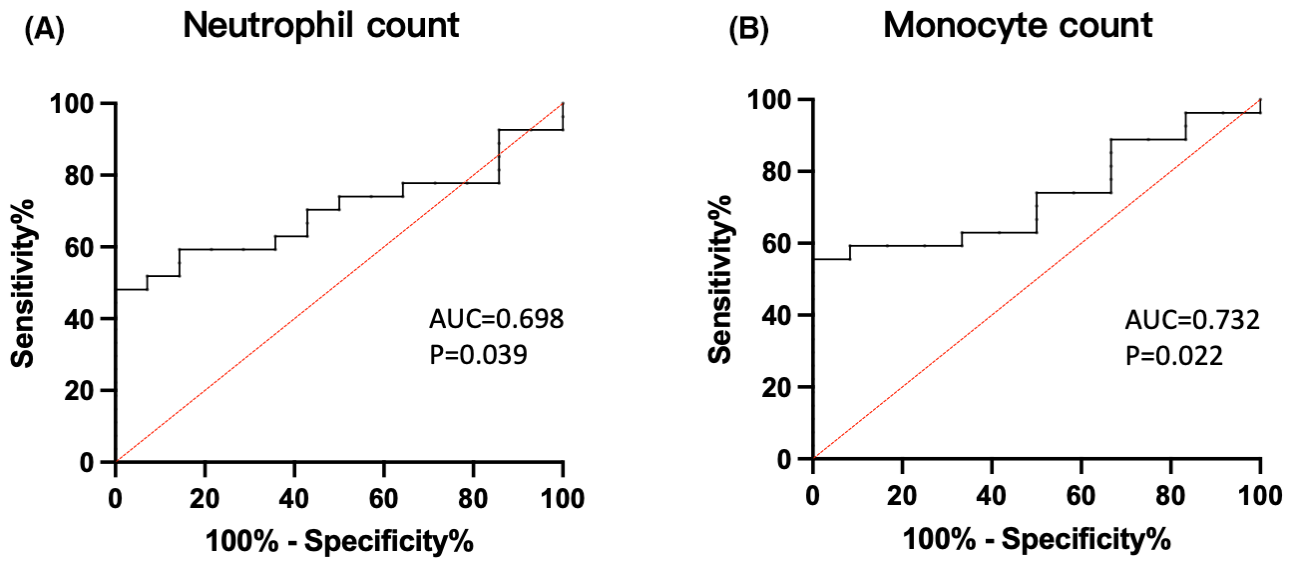
Receiver operating characteristic (ROC) curve of cell counts in predicting liver injury in patients with chronic hepatitis B virus infection. ROC curve of neutrophil count (A) and monocyte count (B).

As shown in Figure 2, 16 CHB patients were defined as undergoing liver fibrosis and inflammation induced by persistent HBV infection, diagnosed via ultrasonography and transaminases. Compared to the group without liver damage, decreased neutrophils and monocytes could predict the occurrence of liver damage among CHB patients, with AUC of 0.698 (*p* = 0.039) and 0.732 (*p* = 0.022), respectively. These results suggest that neutrophils and monocytes, two phagocytic cells of the innate immune system, may be associated with liver damage in chronic HBV infection. The analysis of liver fibrosis and function abnormalities shows no significant differences in leucocyte counts and mitochondrial function, showing supportive evidence for the unified approach to liver damage categorization (data unshown).

## 4 DISCUSSION

HBV is a pernicious chronic pathogen adept at eluding immune surveillance, thus leading to liver immunopathology by triggering abnormal immune reactions, particularly within the adaptive immune system.^15^ Critical to combating HBV infection and mitigating liver injury are the dynamics, scope, intensity, placement and functional capabilities of HBV‐specific immune cells, with a notable emphasis on the roles of CD8^+^ and CD4^+^ T cells.^20^ Despite its profound impact, HBV seems to induce little or no innate immune activation.^21^ In recent decades, growing evidence has demonstrated that inherent immune cells, such as NK, monocytes and macrophages, were enrolled in immune dysfunction within the liver microenvironment of HBV‐related HCC^9^, ^22^

In the present study, we demonstrate decreased neutrophils and monocytes in phagocytic cells, alongside lymphocytes in the peripheral blood of individuals suffering from chronic HBV infection. This study highlights the influence of nucleos(t)ide analogue therapy, prolonged HBV infection and liver damage on the depletion of these vital immune cell populations. In brief, neutrophils and monocytes as the main innate immune cells in peripheral blood have changed in the cohort of chronic HBV infection, even if no end‐stage liver disease is detected.

MM and MMP^low^ are late‐model indicators of mitochondrial function. MM measures the number of protein complexes in the mitochondria's inner membrane respiratory chain, with higher MM levels suggesting an enhanced capacity for ATP production and the dynamics of mitochondrial fusion and division.^14^, ^23^ MMP gauges the voltage difference across the mitochondrial membrane, with certain higher MMP levels, which leads to lower MMP^low^, pointing to heightened ATP synthesis, thus reflecting the cell's metabolic activity.^24^, ^25^, ^26^ In our research, we observed that individuals with chronic HBV infection show a pattern of decreased MM in lymphocytes and monocytes, alongside an increase in MMP^low^%, demonstrating an injured cell metabolic potential and immediately attenuated ATP synthesis. On the contrary, compared to HBV‐negative WBCs, HBV‐positive WBCs displayed a significant elevation in MM across all three cell types, whose potential regulatory mechanisms urgently need to be thoroughly explored. In addition, HBeAg and HBsAg seemed to contribute to MMP^low^% alterations in the neutrophils of lymphocytes. This indicates that the cells are in an active state of ATP supply by mitochondria. Unfortunately, the clinical implications and mechanisms behind HBV's impact on mitochondrial function in WBCs remain undiscovered within this study. However, these observations lead us to invest viral components damaging the mitochondria metabolism of immune cells for future research.

In our investigation, the subjects enrolled had not yet reached the terminal stage of liver disease, including HCC and cirrhosis. However, the data indicate that diminished levels of neutrophils and monocytes are early indicators of liver inflammation and fibrosis. These cells, essential components of the body's immune response, are known to migrate to and accumulate at sites of liver injury. This accumulation, particularly of monocyte‐derived macrophages, is crucial in the progression of liver damage.^27^ This leads to the premise that the observed decrease in these critical immune cells in the bloodstream may reflect their mobilization towards the liver in response to injury, thereby hinting at the onset of liver damage before it progresses to more severe stages. Furthermore, our results of decreased counts due to liver injury and prolonged HBV infection demonstrate neutrophils may also participate in liver injury, thus more experimental confirmation is needed.

Our study inevitably presents some limitations due to the limited cases and clinical phenomena. First, more precise clinical diagnostic thresholds of WBC counts and MM require large‐scale research. Second, for future research, we recommend conducting longitudinal studies to track changes in mitochondrial function and leucocyte counts over time in patients with HBV infection. This approach would provide a deeper understanding of the dynamics of these changes. Further experiments are needed to investigate the underlying mechanisms by which HBV affects leucocyte mitochondrial function.

In summary, our investigation indicates that decreased WBC counts and mitochondrial dysfunction based on feasible flow cytometry have been altered significantly in the cohort of chronic HBV infection, although they have not yet progressed to end‐stage liver disease. Decreased neutrophils and monocytes of non‐specific phagocytic cells in the peripheral bloodstream manifest as good prognostic indicators for liver damage by persistent HBV infection. Moreover, the mitochondrial function of leucocytes is significantly affected by the presence of intracellular HBV, HBeAg and HBsAg of the viral components. Anti‐viral therapy by NA(s) drug, prolonged infection and liver injury seem to primarily affect the number of WBCs. More research in the future is needed to focus on discovering potential regulatory mechanisms contributing clinical application of WBC counts combined with mitochondrial dysfunction in HBV infection.

## AUTHOR CONTRIBUTIONS

**Ruo‐Ran Zhou:** Conceptualization (equal); methodology (equal); writing – original draft (equal). **Ya‐Hui Song:** Investigation (equal). **Cheng‐Yu Xu:** Investigation (equal). **Ying‐Ying Zhang:** Investigation (equal). **Xiang‐Wei Wu:** Investigation (equal). **Lu Zhang:** Investigation (equal). **Xi‐Ni Luo:** Formal analysis (equal). **Han Zhao:** Formal analysis (equal). **Ming‐Ming Liu:** Resources (equal). **Jun‐Chi Xu:** Resources (equal). **Lin Wang:** Funding acquisition (equal); project administration (equal); writing – review and editing (equal). **Zu‐Tao Chen:** Resources (equal). **Qing‐Zhen Han:** Conceptualization (equal); funding acquisition (equal); methodology (equal); project administration (equal); supervision (equal); writing – original draft (equal); writing – review and editing (equal).

## FUNDING INFORMATION

The present study was financially supported by the Suzhou Science and Technology Planning Project (grant nos SLT201921, SZM2021011, SZM2021018 and SKY2022091).

## CONFLICT OF INTEREST STATEMENT

The authors confirm that there are no conflicts of interest.

## Supporting information


Figure S1.

## Data Availability

Data available on request from the authors.
