# Supplementary material for: Altered counts and mitochondrial mass of peripheral blood leucocytes in patients with chronic hepatitis B virus infection
Source: J Cell Mol Med. 2024 Jun 18;28(12):e18440. doi: 10.1111/jcmm.18440 (PMC11187856; doi:10.1111/jcmm.18440)
Supplement: Supplementary file 1 — Figure S1. [file JCMM-28-e18440-s001.pdf]

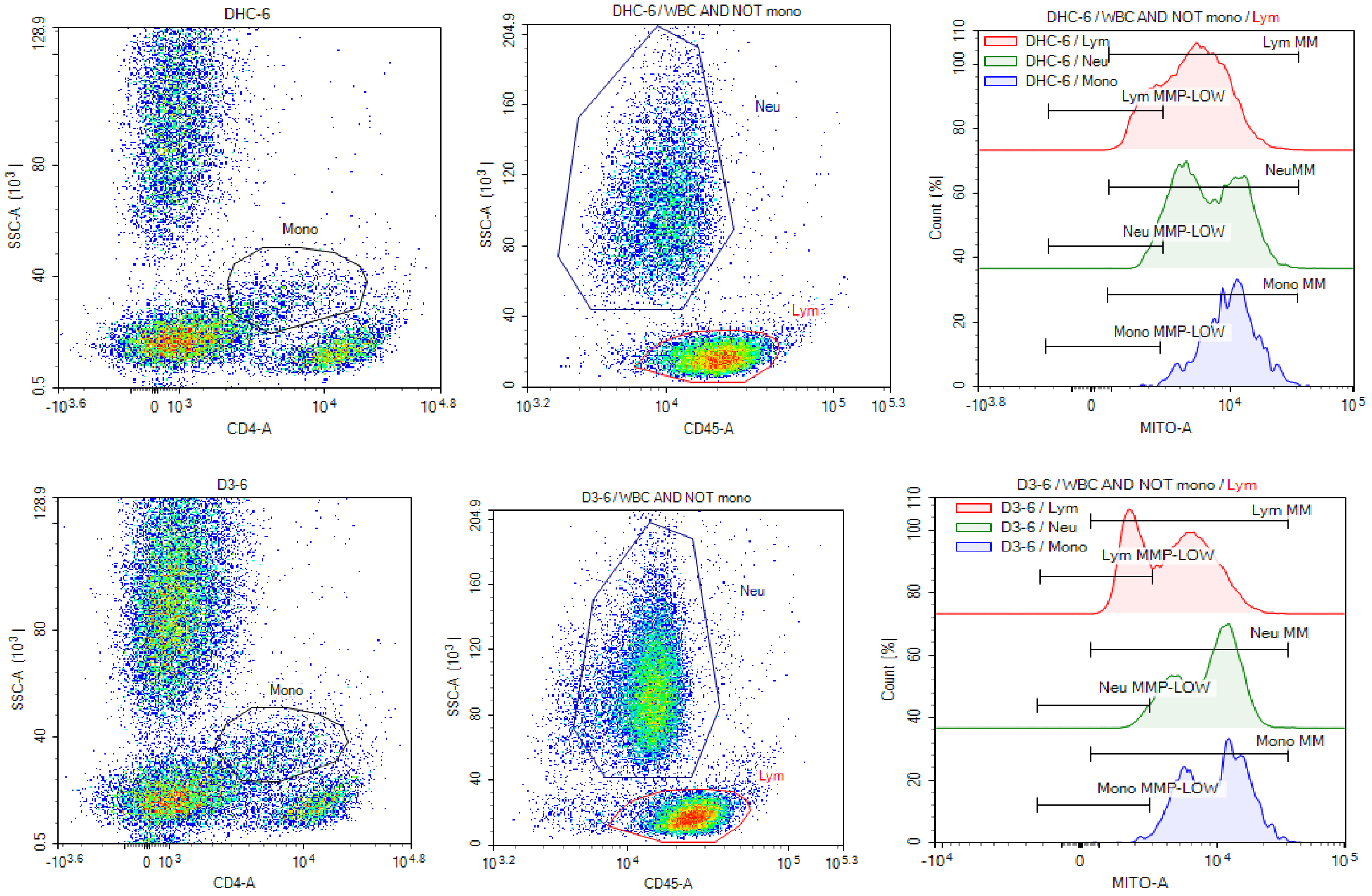

Figure S1 Mitochondrial mass of WBCs detected by flow cytometry.
DHC-6 and D3-6 refer to sample numbers.
